# Efficacy and safety of pyrotinib in the treatment of HER2-positive liver metastatic advanced breast cancer

**DOI:** 10.3389/fonc.2025.1527277

**Published:** 2025-04-08

**Authors:** Yongxia Li, Yong Li, Taolang Li, Mingyuan He, Jianying Chang, Hui Cao, Daiqin Luo, Junyuan Lv, Yunbi Zou, Yuyan Zheng, Li Ran, Feiyue Yang, Li Huang, Xiaoming Cheng

**Affiliations:** ^1^ Department of Oncology, The Affiliated Hospital of Guizhou Medical University, Guiyang, Guizhou, China; ^2^ Department of Breast Oncology, The Affiliated Cancer Hospital of Guizhou Medical University, Guiyang, Guizhou, China; ^3^ Department of Oncology, Guizhou Provincial People’s Hospital, Guiyang, Guizhou, China; ^4^ Department of General Surgery & Thyroid and Breast Surgery, Affiliated Hospital of Zunyi Medical University, Zunyi, Guizhou, China; ^5^ Department of Oncology, Hospital of Guizhou Panjiang Coal Power Group Co., LTD., Panzhou, Guizhou, China; ^6^ Teaching and Research Section of Oncology, Guizhou Medical University, Guiyang, Guizhou, China

**Keywords:** breast cancer, pyrotinib, HER2-positive, liver metastasis, efficacy and safety

## Abstract

**Background:**

This study aimed to evaluate the efficacy and safety of pyrotinib in the treatment of HER2-positive breast cancer patients with and without liver metastasis.

**Methods:**

A retrospective analysis was conducted on 91 patients with HER2-positive advanced breast cancer, who were treated with pyrotinib between March 2019 and April 2022. The patients were categorized into two groups based on the presence or absence of liver metastases, and their overall survival (OS), progression-free survival (PFS), and their response to pyrotinib were compared. Adverse effects in the patients were analyzed to assess the safety of pyrotinib.

**Results:**

The cohort include 29 patients with liver metastasis and 62 without. The median overall survival was significantly shorter in the liver metastasis group (15.8 months) than that in the non-liver metastasis group (31.4 months, P = 0.0036). A statistically significant difference was observed in the median PFS between the liver metastasis and the non-liver metastasis groups (8.7 vs. 18.4 months) (P = 0.0272). Univariate analysis revealed that patients with younger age (<60 years) (P < 0.0001), negative progesterone receptor expression (P = 0.0028), higher Ki67 expression levels (P < 0.0001), and absence of lymph node metastasis (P < 0.0001) were more likely to benefit from pyrotinib treatment. Comparative analysis between groups showed significantly higher incidence rates of anemia (58.6% vs. 40.3%) and elevated aspartate transaminase level (31.0% vs 8.1%) in the liver metastasis group compared to the non-liver metastasis (P < 0.05).

**Conclusions:**

Pyrotinib-based therapy is efficacious and safe for patients with HER2-positive advanced breast cancer with liver metastases, while further large-scale clinical trials are warranted to validate these results.

## Introduction

Cancer represents a significant global public health challenge, accounting for a substantial proportion of worldwide mortality. Notably, breast cancer constituted almost one-third of all cancers diagnoses among women in 2022 (1, 2). The human epidermal receptor factor 2 (HER2), a transmembrane protein comprising four receptor tyrosine kinases, has been identified as a critical oncogenic driver through its prominent overexpression in various malignancies, facilitating oncogenic transformation and tumorigenesis (3). HER2-positive breast cancer, characterized by its aggressive clinical behavior and elevated recurrence rates, accounts for approximately 15-20% of all breast cancer subtypes and is associated with unfavorable prognosis (4–6). Consequently, HER2-targeted therapies have emerged as an essential therapeutic strategy for managing this breast cancer subtype (7).

Pyrotinib, an innovative tyrosine kinase inhibitor, has demonstrated significant anti-tumor activity against HER2-positive metastatic breast cancer (8–10). Following its conditional approval in China in 2018 for the treatment of HER2-positive metastatic or advanced breast cancer (11), pyrotinib has been incorporated into clinical guidelines. Specifically, the Guidelines for the Diagnosis and Treatment of Breast Cancer (2021) issued by the Chinese Anti-Cancer Association recommend pyrotinib in combination with capecitabine for HER2-positive advanced breast cancer. Clinical evidence from a phase II study revealed that pyrotinib combined with capecitabine significantly prolonged median progression free survival (PFS) in HER2-positive metastatic breast cancer patients compared to lapatinib plus capecitabine regimens (12).

Metastasis to vital organs remains the primary determinant of mortality in breast cancer, with metastatic heterogeneity across different organs systems contributing to varied prognostic outcomes and therapeutic responses (13, 14). Among metastatic sites, the liver represents the most common destination for solid tumor dissemination and ranks as the third most frequent site for breast cancer metastasis (15). Clinical data indicated that untreated breast cancer patients with liver metastasis have a survival period of merely 4-8 months, with a 5-year overall survival (OS) rate of 8.5% (16, 17). The therapeutic landscape for HER2-positive breast cancer with liver metastasis remains particularly challenging, with limited effective treatment options currently available. In this study, we stratified HER2-positive advanced breast cancer patients based on liver metastasis status to systematically evaluate the efficacy and safety profile of pyrotinib-based regimens. Our findings are anticipated to contribute to the development of more effective and safer clinical treatment options for HER2-positive breast cancer with liver metastasis, while simultaneously providing valuable data to enhance our understanding of the heterogeneity associated with breast cancer liver metastasis.

## Materials and methods

### Study population

This multicenter, real-world retrospective study was conducted in compliance with the ethical principles outlined in the Declaration of Helsinki (2013 revision). The study protocol was reviewed and approved by the Ethics Committee of The Affiliated Cancer Hospital of Guizhou Medical University (approval number: FZ-202105130). Due to the retrospective nature of the study, the requirement for individual informed consent was waived by the Ethics Committee.

A total of 91 patients with HER2-positive advanced breast cancer, treated across four medical centers in Guizhou, China, from March 2019 to April 2022, were included in the analysis. Follow-up data were collected until February 2023. The patient distribution across the four participating oncology departments was as follows: The Affiliated Cancer Hospital of Guizhou Medical University (58 patients), Guizhou Provincial People’s Hospital (21 patients), the Affiliated Hospital of Zunyi Medical University (5 patients), and the Hospital of Guizhou Panjiang Coal Power Group Co., LTD. (7 patients).

Patients were enrolled based on the following inclusion criteria: (I) Female patients aged ≥ 18 and ≤ 70 years with HER2-positive breast cancer; (II) HER2 positivity, defined as an immunohistochemistry score of 3+ or 2+ combined with *HER2* gene amplification confirmed by fluorescence *in situ* hybridization) (18); (III) Eastern Cooperative Oncology Group (ECOG) performance status of 0–2; (IV) Presence of at least one measurable lesion according to the Response Evaluation Criteria in Solid Tumors (RECIST) version 1.1 (19); (V) Adequate bone marrow, hepatic, renal, and cardiac function, with normal left ventricular ejection fraction; (VI) A life time expectancy of more than 3 months to allow completion of treatment and follow-up; (VII) Completion of at least two cycles of treatment, either with single pyrotinib monotherapy or combination therapy.

The exclusion criteria were as follows: (I) pregnancy or lactation; (II) Severe comorbidities affecting the endocrine, respiratory, circulatory, or digestive system; (III) History of drug absorption or metabolism disorders; (IV) Diagnosis of inflammatory breast cancer or bilateral breast cancer; (V) Presence of a second primary malignant tumor.

### Data collection

Demographic and baseline clinical characteristics were collected, including age, prior treatment history, presence of distant metastasis (brain, bone, or lung), lines of pyrotinib therapy, and combination chemotherapy regimens. Additionally, hormone receptors (HR) including estrogen receptor (ER) and progesterone receptor (PR) status, Ki67 expression levels, and lymph node metastasis status were recorded for survival and univariate analyses.

### Treatment and outcomes evaluation

Patients received oral pyrotinib (Jiangsu Hengrui Pharmaceuticals Co., LTD) at a baseline dose of 400mg daily, with dose adjustments permitted based on adverse effects (AEs). Tumor response was assessed every 6 weeks during treatment and every 3 months post-treatment using comprehensive imaging, in accordance with RECIST v1.1. Overall Survival (OS) is defined as the time from the first administration of pyrotinib to the last follow-up visit or death. Progression-free survival (PFS) refers to the time from treatment initiation to the first documented disease progression.

Tumor response was categorized according to the following criteria. Complete response (CR): disappearance of all target lesions; partial response (PR): ≥30% reduction in the sum of the maximum diameter of target lesions; progressive disease (PD): ≥20% increase in the sum of the maximum diameters of target lesions or the appearance of new lesions; stable disease (SD): changes in the sum of the maximum diameters of target lesions that did not meet the criteria for PR or PD (19). The objective response rate (ORR) was calculated as (CR + PR)/total lesions×100%; the disease control rate (DCR) as (CR + PR + SD)/total lesions×100%; and the clinical benefit rate (CBR) as (CR + PR + SD)/total lesions×100%. Adverse events (AEs) were evaluated using the National Cancer Institute Common Terminology Criteria for Adverse Events version 5.0 (CTCAE5.0).

### Statistical analysis

Data were analyzed using SPSS version 26.0. All statistical tests were two-sided, with a significance threshold of P <0.05. Continuous variables were expressed as median ± standard error of the mean and were compared using Student’s *t*-test. Categorical variables were reported as frequencies (percentages) and compared using the Chi-square test or Fisher’s exact test, as appropriate. Univariate analysis of clinical variables was performed using the Log-rank method. Median OS and PFS were estimated using Kaplan-Meier curves, and survival curves were plotted using GraphPad Prism version 8.0.

## Results

### Baseline characteristics

**Figure 1:**
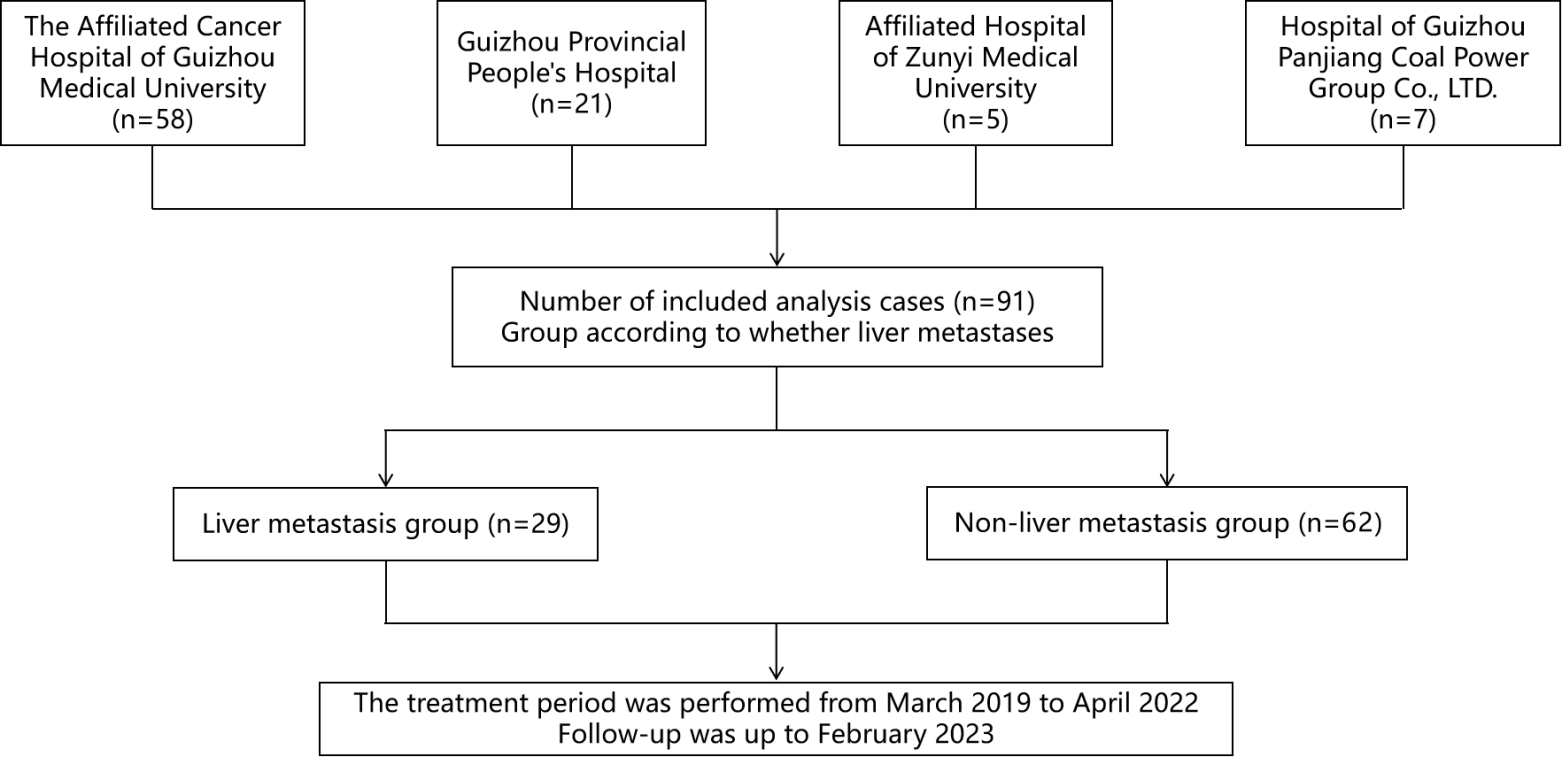
Flow diagram of this study.

Among the 91 patients included in this study, 29 (31.9%) were diagnosed with liver metastases prior to pyrotinib treatment, while 62 (68.1%) had no liver metastases (non-liver metastasis group). The study design flow diagram is illustrated in **Figure 1**. Comparative analysis of demographic and baseline characteristics between the two groups is summarized in **Table 1**. The mean age of the liver metastasis group was 49.28 ± 1.39 years, compared to 52.27 ± 1.12 years in the non-liver metastasis group. In the liver metastasis group, 26 patients (89.7%) received pyrotinib in combination with chemotherapy agents (vinorelbine, taxanes, capecitabine, or pharmorubicin), while 55 patients (88.7%) in the non-liver metastasis group received similar combination therapy. No statistically significant differences were observed in demographic or baseline characteristics between the two groups (P > 0.05).

**Table 1:** Demographic and baseline characteristics of patients in different groups.

Characteristic	Liver metastasis (N=29)	Non-liver metastasis (N=62)	*P* value
Age (years), n (%)			0.054
< 60	28 (96.6)	50 (80.7)	
≥ 60	1 (3.4)	12 (19.3)	
With adjuvant or neoadjuvant therapy in newly diagnosed patients, n (%)			0.924
Yes	17 (58.6)	37 (59.7)	
No	12 (41.4)	25 (40.3)	
Previously received anti-HER2 therapy, n (%)			1.000
Yes	25 (86.2)	54 (87.1)	
No	4 (13.8)	8 (12.9)	
With metastasis in brain, bone, or lung, n (%)			1.000
Yes	25 (86.2)	52 (83.9)	
No	4 (13.8)	10 (16.1)	
Lines of pyrotinib, n (%)			0.623
≤ 2	7 (24.1)	19 (30.6)	
> 2	22 (75.9)	43 (69.4)	
Combined chemotherapy, n (%)			1.000
Yes	26 (89.7)	55 (88.7)	
No	3 (10.3)	7 (11.3)	

### Efficacy

**Figure 2:**
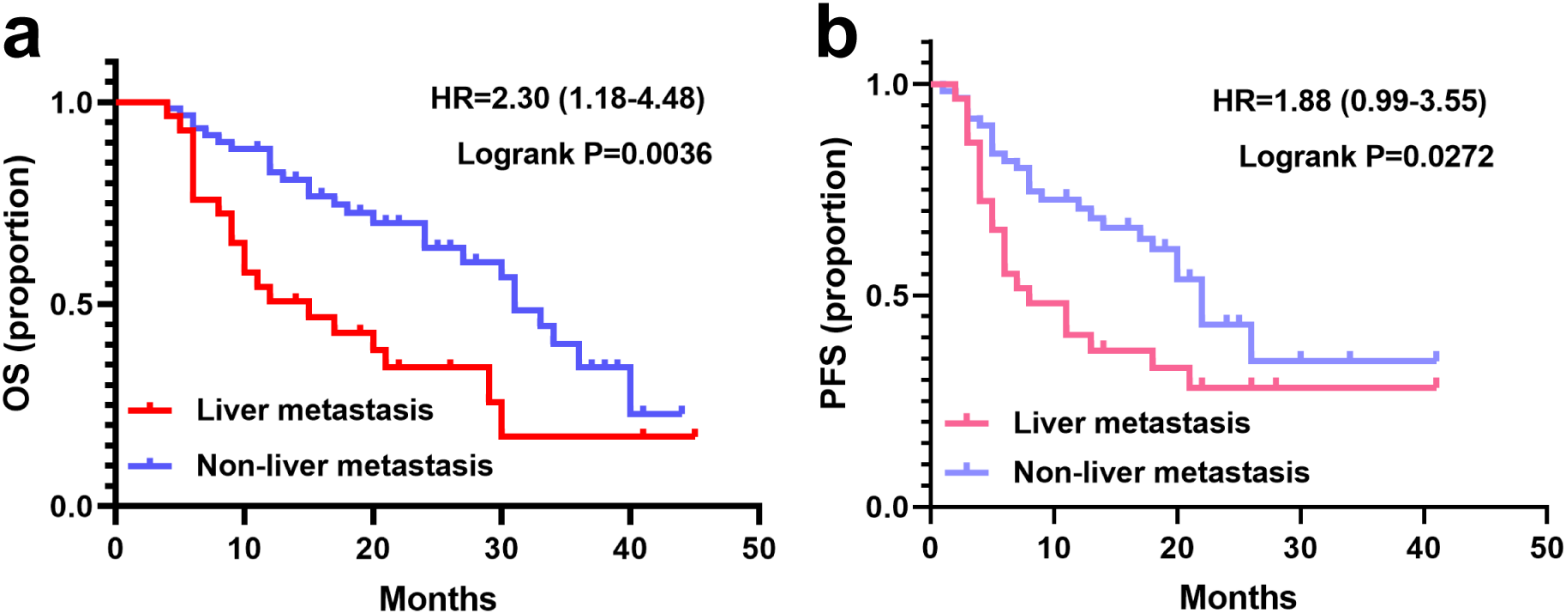
Survival curves of patients with or without liver metastasis. **(a)** OS in the liver metastasis (N=29) and non-liver metastasis groups (N=62). **(b)** PFS with the liver metastasis and non-liver metastasis groups.

All patients had completed at least four cycles of treatment. The median follow-up durations were 26.4 months (95%CI: 20.8 - 32.0) for the liver metastasis group and 25.5 months (95%CI: 17.4 - 33.6) for the non-liver metastasis group (P > 0.05). Survival curves for both groups are presented in **Figure 2**. The median OS in the liver metastasis group was 15.8 months (95%CI: 6.5 - 25.1), significantly shorter than that of the non-liver metastasis group (31.4 months; 95%CI: 27.3 - 35.5; P = 0.0036). The median PFSs of liver metastasis group and non-liver metastasis group were 8.7 (95%CI: 1.7 - 15.7) months vs 18.4 (95%CI: 11.6 - 25.2) months (P = 0.0272).

**Figure 3:**
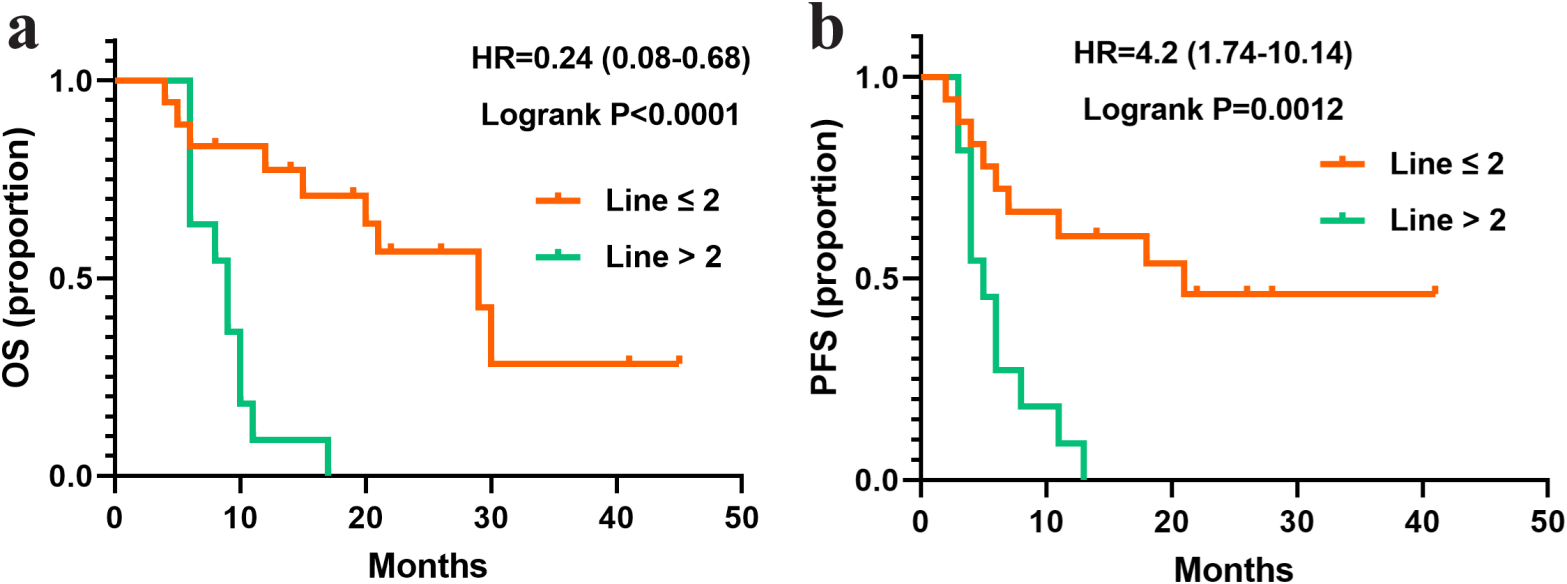
Survival curves of patients with liver metastasis and treated with different lines of pyrotinib. **(a)** OS in the patients treated with pyrotinib in line ≤ 2 (N=18) or >2 (N=11). **(b)** PFS in the patients treated with pyrotinib in line ≤ 2 (N=18) or >2 (N=11).

To identify subgroups of HER2-positive breast cancer patients with liver metastases (n=29) who may derive greater benefit from pyrotinib treatment, a subgroup analysis was performed based on HR status (ER, PR, or combined), Ki67 expression levels, and lymph node metastasis status. Survival analysis revealed no significant differences in median OS or PFS across subgroups defined by ER/PR expression, Ki67 expression levels, and lymph node metastasis (P > 0.05). However, patients with negative PR expression exhibited a trend toward longer median OS (20 months), although this difference did not reach statistical significance (P = 0.051). Notably, patients who received pyrotinib as a first- or second-line treatment (line ≤ 2) demonstrated significantly longer OS compared to those who received is as a later-line therapy (line > 2) (P < 0.0001; **Figure 3**).

### Clinical response

**Figure 4:**
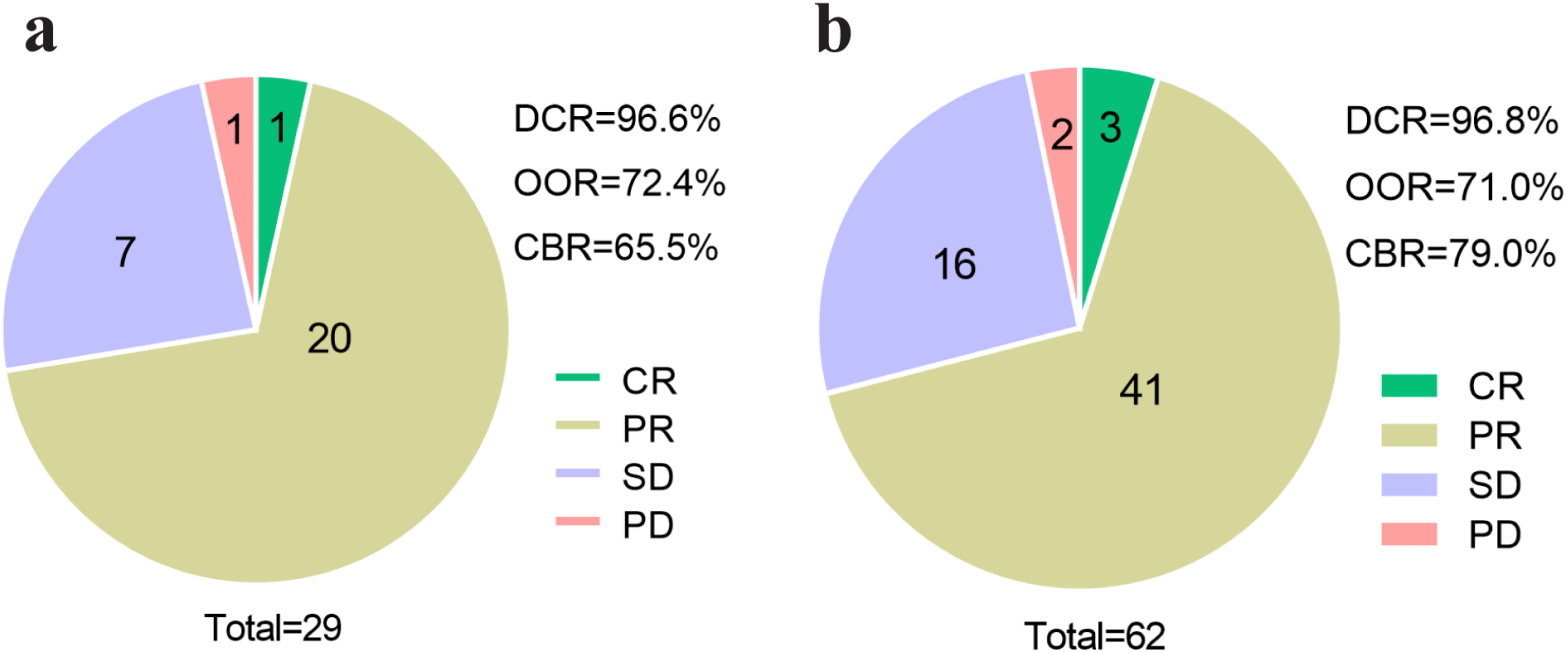
Efficacy assessment of pyrotinib in patients with or without liver metastasis. **(a)** Efficacy assessment of patients of liver metastasis group (N=29), **(b)** efficacy assessment of patients of non-liver metastasis group (N=62). CR, complete response; PR, partial response; SD, stable disease; PD, progressive disease; DCR, disease control rate; ORR, objective response rate; CBR, clinical benefit rate.

The efficacy of pyrotinib was evaluated in both liver metastasis and non-liver metastasis groups, with results summarized in **Figure 4**. In the liver metastasis group, treatment outcomes included 1 case of CR, 20 cases of PR, 7 cases of SD, and 1 case of PD. The DCR, ORR, and CBR were 96.6% (28/29), 72.4% (21/29) and 65.5% (19/29), respectively. In the non-liver metastasis group, outcomes included 3 cases of CR, 41 cases of PR, 16 cases of SD, and 2 cases of PD, with corresponding DCR, ORR, and CBR of 96.8% (60/62), 71.0% (44/62) and 79.0% (49/62), respectively. No statistically significant differences in clinical response rates were observed between the two groups (P > 0.05).

To further refine the patient population most likely benefit from pyrotinib therapy, patients were stratified into responders (CR+PR) and non-responders (SD+PD) for univariate analysis. As shown in **Table 2**, patients with younger age (<60 years; P < 0.0001), negative PR expression (P = 0.0028), higher Ki67 expression levels (P < 0.0001), and absence of lymph node metastasis (P < 0.0001) were more likely to benefit from pyrotinib treatment.

**Table 2:** Univariate analysis of factors associated with the response to pyrotinib treatment.

Variables	Responders (N=65)	Non-responders (N=26)	OR (95% CI)	*P* value
Age (years), n (%)				<0.0001
< 60	56 (86.2)	22 (84.6)		
≥ 60	9 (13.8)	4 (15.4)	0.17 (0.09-0.29)	
ER, n (%)				0.1744
Positive	29 (44.6)	10 (38.5)		
Negative	36 (55.4)	16 (61.5)	0.75 (0.49-1.13)	
PR, n (%)				0.0028
Positive	24 (36.9)	7 (26.9)		
Negative	41 (63.1)	19 (73.1)	0.52 (0.33-0.79)	
HR, n (%)				0.75
Positive	34 (52.3)	16 (61.5)		
Negative	31 (47.7)	10 (38.5)	0.94 (0.62-1.41)	
Ki67, n (%)				<0.0001
< 30%	9 (13.8)	9 (34.6)		
≥ 30%	56 (86.2)	17 (65.4)	4.06 (2.48-7.00)	
Lymph node metastasis, n (%)				<0.0001
Yes	10 (15.4)	6 (23.1)		
No	55 (84.6)	20 (76.9)	0.21 (0.12-0.36)	

### Safety

AEs in both groups are summarized in **Table 3**. The most common AEs in the liver metastasis group were diarrhea (93.1%), emesis (65.5%), and anemia (58.6%). In the non-liver metastasis group, the most frequent AEs were diarrhea (93.5%), leukopenia (41.9%), anemia (40.3%), and nausea (40.3%). Diarrhea was the most severe AE in both groups, with grade 3-4 diarrhea occurring in 41.4% of the liver metastasis group and 29.0% of the non-liver metastasis group. Compared to the non-liver metastasis group, the liver metastasis group exhibited a significantly higher incidence of anemia (58.6% vs 40.3%, P < 0.05) and elevated aspartate transaminase (AST) levels (31.0% vs 8.1%, P < 0.05; **Table 3**). No statistical differences were noted in the incidence of other AEs between the two groups.

**Table 3:** AEs of patients in different groups.

AE	Liver metastasis (N = 29) (n, %)		Non-liver metastasis (N = 62) (n, %)	
	Any grade	Grade 3-4	Any grade	Grade 3-4
Diarrhea	27 (93.1)	12 (41.4)	58 (93.5)	18 (29.0)
Emesis	19 (65.5)	0	10 (16.1)	0
Anemia	17 (58.6)*	2 (6.9)	25 (40.3)	0
Leukopenia	17 (58.6)	5 (17.2)	26 (41.9)	7 (11.3)
Nausea	16 (55.2)	0	25 (40.3)	0
Neutropenia	15 (51.7)	4 (13.8)	22 (35.5)	4 (6.5)
AST increased	9 (31.0)*	0	5 (8.1)	0
ALT increased	6 (20.7)	0	6 (9.7)	0
Thrombocytopenia	6 (20.7)	1 (3.4)	5 (8.1)	0
Creatinine increased	5 (17.2)	0	11 (17.7)	0
Hand-foot syndrome	4 (13.8)	0	8 (12.9)	0

AE, adverse event; ALT, alanine transaminase; AST, aspartate transaminase. *P < 0.05.

## Discussion

This retrospective study aimed to evaluate and compare the real world efficacy and safety of pyrotinib in the treatment of HER2-positive breast cancer patients with or without liver metastasis. Our findings demonstrate that pyrotinib-based regimens are both effective and safe for HER2-positive advanced breast cancer patients with liver metastases, with anemia and liver injury being the primary adverse effects, which were generally manageable and clinically tolerable.

The efficacy of pyrotinib in HER2-positive advanced breast cancer has been previously established. For instance, a phase II study reported an ORR of 78.5% in 65 patients treated with pyrotinib (12). Similarly, another phase II clinical trial involving 40 HER2-positive metastatic breast cancer patients demonstrated a DCR of 97.5%, ORR of 50.5%, and CBR of 75.5% with pyrotinib-based therapy (20). In our study, the DCR, ORR, and CBR in HER2-positive breast cancer patients with liver metastasis were 96.6%, 72.4%, and 65.6%, respectively, with no significant differences compared to the non-liver metastasis group. These results suggest that pyrotinib remains effective even after liver metastases. Furthermore, our analysis identified that younger patients (<60 years), those with negative PR expression, higher Ki67 expression levels, and absence of lymph node metastasis were more likely to respond to pyrotinib treatment. This aligns with previous findings indicating that the patients aged ≥ 65 but < 70 years had higher ORR and CBR compared to those aged ≥ 70 years (21). Additionally, a published study has shown that patients with negative hormone receptor status (ER, PR, or androgen receptor) are more likely to achieve total pathologic (CR) compared to those with positive receptor status (22). However, conflicting results have been reported regarding Ki67 expression, with one study showing lower pathologic CR rates in patients with Ki67 ≥30% compared to those with Ki67 <30% when treated with pyrotinib, trastuzumab, and chemotherapy (23). These discrepancies may be attributed to differences in treatment regimens, including the use of trastuzumab or varying chemotherapy agents (24), warranting further investigation to establish precise treatment guidelines.

In our study, a significant difference in median PFS was observed between the non-liver metastasis group (18.4 months) and the liver metastasis group (8.7 months). This contrasts with a previous study reporting median PFS of 8.7 and 12.3 months in patients with and without liver metastases respectively (P = 0.172) (25). However, another retrospective study involving 172 HER2-positive metastatic breast cancer identified visceral metastasis as an independent prognostic factor for PFS (8.40 vs. 23.70 months; P = 0.0138) (26). Additionally, liver and/or lung metastases have been shown to adversely affect PFS in HER2-positive advanced breast cancer patients treated with pyrotinib (20). Our data also revealed significantly shorter OS in the liver metastasis group (15.8 moths) compared to the non-liver metastasis group (31.4 months; P = 0.0036). These findings suggest that while pyrotinib may provide short-term benefits for HER2-positive breast cancer patients with liver metastases, liver metastasis alone is not the sole determinant of PFS and OS. Further research with larger sample size and extended follow-up periods is needed to identify additional prognostic factors.

The safety profile of pyrotinib in HER2-positive advanced breast cancer has been well-documented, with diarrhea, anemia, emesis, nausea, and leukopenia being the most commonly reported AEs (27–30). In our study, diarrhea and liver injury were the predominant AEs in patients with liver metastasis. Fortunately, these adverse effects were generally manageable with supportive care, such as antidiarrheal agents and hepatoprotective medications (31, 32). Once resolved, pyrotinib remains a promising therapeutic option with favorable efficacy and safety for HER2-positive breast cancer with liver metastases.

This study has several limitations. First, as a retrospective analysis, it is subject to potential information bias due to missing clinical data. Second, the sample size of 91 patients may limit the generalizability of the findings, and larger studies are needed to validate these results. Third, the impact of additional clinical factors, such as brain or lung metastases and combined radiotherapy, on treatment response should be investigated in future large-scale clinical trials. Addressing these limitations through further research will enhance our understanding of pyrotinib-based therapy and improve patient outcomes.

In conclusion, the pyrotinib-based therapy demonstrated efficacy in treating HER2-positive advanced breast cancer with liver metastasis, with manageable and tolerable adverse effects. These findings support the use of pyrotinib as a viable treatment option for this patient population, although further studies are warranted to optimize treatment strategies and improve prognostic outcomes. In the future, research efforts may focus on identifying potential biomarkers to predict the efficacy of pyrotinib in HER2-positive breast cancer. For instance, investigating the role of peripheral blood mononuclear cells, gut microbiota, or metabolic products as predictive indicators of treatment response could provide valuable insights (33).

## Data Availability

The original contributions presented in the study are included in the article/supplementary material. Further inquiries can be directed to the corresponding authors.
